# Hemodynamic actions of insulin: beyond the endothelium

**DOI:** 10.3389/fphys.2013.00389

**Published:** 2013-12-24

**Authors:** David Montero

**Affiliations:** Applied Biology Department, Institute of Bioengineering, Miguel Hernandez UniversityElche, Spain

**Keywords:** insulin, hemodynamic action, endothelium, vascular smooth muscle, capillary recruitment

## Brief historical overview

Insulin's hemodynamic actions are being progressively unraveled over the last two decades (Baron, 1994; Clark, 2008). Originally, Baron et al (Laakso et al., 1990) reported insulin-mediated increase in total blood flow, suggesting an associated higher delivery of nutrients and insulin to target tissues. Nonetheless, the physiological meaning of that extensive hemodynamic action of insulin is controversial, given that lower insulin concentrations than those required to increase total blood flow, stimulate muscle glucose uptake (Yki-Jarvinen and Utriainen, 1998; Zhang et al., 2004). Afterward, a distinct vascular action of insulin was observed, which resulted in capillary recruitment (Rattigan et al., 1997). Such capillary recruitment occurs at physiological concentrations of insulin, precedes increases in total blood flow and is directly correlated to glucose uptake in skeletal muscle (Rattigan et al., 1997, 1999; Coggins et al., 2001). Provided that insulin transcapillary transport plausibly involves a predominant non-saturable process in skeletal muscle (Steil et al., 1996; Hamilton-Wessler et al., 2002; Majumdar et al., 2012), insulin-mediated capillary recruitment emerges as a crucial action of insulin, coupling vascular and metabolic physiology. An integrative pathophysiological view of the relationships between hemodynamic and metabolic functions of insulin has been presented in detail in previous reviews (Muniyappa et al., 2007; Clark, 2008; Muris et al., 2013). Likewise, it has been recognized a similar vascular and metabolic insulin-signaling via insulin receptor/phosphatidylinositol 3-kinase (PI3K) pathways (Kim et al., 2006; Kubota et al., 2011), which are present in both endothelium and vascular smooth muscle (VSM), leading to nitric oxide synthase (NOS) activation, increased nitric oxide (NO) production and subsequent vasodilation (Zeng and Quon, 1996; Anfossi et al., 2002). Most previous research in this field has focused on insulin actions in and/or through the endothelium, assumed as the preponderant sensor/effector organ underlying the vascular effects of insulin (Muniyappa et al., 2007; Kubota et al., 2011). However, recently increased animal and human evidence suggests a leading hemodynamic role for the direct insulin action in VSM (Rossi et al., 2005; Newman et al., 2009; Montero et al., 2013a,c), thus requiring to reconsider widely held conceptions in this field.

## Conflicting findings on endothelium-mediated actions of insulin

Taking the generalized view that couples endothelial dysfunction with insulin resistance (Kim et al., 2006) and considering insulin-mediated capillary recruitment as a rate-limiting step for glucose uptake (Rattigan et al., 1999; Chiu et al., 2008), the fact remains that the targeted disruption of the insulin receptor in the endothelium does not alter glucose homeostasis (Vicent et al., 2003; Duncan et al., 2008; Rask-Madsen et al., 2010). Conversely, mice with endothelial cell-specific knockout of insulin receptor substrate 2 (IRS-2), a signaling intermediate in the activation of PI3K, do indeed show impaired insulin-mediated capillary recruitment and muscle glucose uptake (Kubota et al., 2011). Notwithstanding the lack of satisfactory explanations for these conflicting reports, insulin signaling in the endothelium is considered to determine the access of glucose and insulin to target tissues (Kolka and Bergman, 2013). Yet, several issues might be pondered before to confirm endothelial-mediated insulin action(s) as the crucial hemodynamic one(s) impacting on glucose homeostasis. First, the aforementioned study linking vascular and metabolic impairment of insulin action (Kubota et al., 2011) determined IRS-2 expression in the endothelium, adipose tissue, liver and skeletal muscle, however, no such data were displayed in VSM, which might be a desirable proof of specificity of impaired insulin- and endothelial-mediated capillary recruitment. Second, allowing that insulin signaling in the endothelium increases NO availability, its contribution to glucose homeostasis may be, to some extent, independent of glucose and insulin delivery, since NOS inhibition and reduced NO levels has been associated with ~35% attenuation of contraction-stimulated glucose uptake without altering the increase in capillary recruitment (Ross et al., 2007). Third, increased, rather than decreased, insulin signaling in the endothelium predisposes to systemic insulin resistance (Tsuchiya and Accili, 2013). Fourth, insulin may prompt NO-independent vasodilation through mechanisms involving the activation of potassium channels in conduit arteries (Hasdai et al., 1998; Izhar et al., 2000) and arterioles (McKay and Hester, 1996; Colantuoni et al., 2005) and removal of the endothelium does not modify this response (Hasdai et al., 1998; Izhar et al., 2000). Furthermore, under ischemic conditions, insulin induces arteriolar vasodilation and capillary recruitment by the activation of potassium channels, which subsequently may result in the release of NO via increased shear stress on endothelial cells, therein sustaining arteriolar vasodilation and capillary recruitment (Bertuglia and Colantuoni, 1998; Colantuoni et al., 2005). Interestingly, arginine analogs commonly used to determine NO contribution in vascular research, have been shown to inhibit potassium channel-dependent arteriolar vasodilation (Kontos and Wei, 1996), which may cast reasonably doubt on previous conclusions (Vincent et al., 2003). Fifth, terminal arterioles, which are more sensitive to insulin than larger blood vessels (i.e., conduit and resistance arteries, first- and second-order arterioles (Porter et al., 1997; Oltman et al., 2000; Zhang et al., 2004)) and are proposed to play a decisive role in capillary recruitment, do not rely on NO-dependent but in potassium channel-dependent vasodilation (McKay and Hester, 1996; Oltman et al., 2000). Taken together, it can be presumed that pivotal hemodynamic actions of insulin such as capillary recruitment might be mediated, primarily or not, by physiological effectors other than endothelial cells.

## Evidence of VSM-mediated hemodynamic actions of insulin

As a key physiological variable in the regulation of microvascular perfusion, the study of vasomotion, i. e., the spontaneous rhythmic change in arteriolar diameter, has provided relevant insight on the hemodynamic actions of insulin (Clark, 2008). Through the analysis of the effects of vasomotion on perfusion oscillations, Rossi et al (Rossi et al., 2005) discovered that locally administered insulin specifically increases VSM (myogenic) activity in the skin microcirculation of healthy adults. In contrast, De Jongh et al. (2004) reported that, in healthy adults, hyperinsulinemia increased intramuscular microvascular oscillations attributed to endothelial and neurogenic activity. The reasons for this discrepancy are probably not related to differences in vascular bed responsiveness, since there is no evidence of distinct vascular effects of insulin in skin and muscle (Meijer et al., 2012). Instead, these equivocal findings may be explained by differences in the expression of results. In fact, Newman et al. (2009) found higher myogenic activity in the muscle microvasculature during hyperinsulinemia when the amplitudes of each oscillation interval were normalized by the oscillatory activity of the entire spectrum, effectively taking into account differences in the oscillatory signal strength between experiments (Bracic and Stefanovska, 1998). Importantly, the addition of α-methylserotonin, which is a peripheral vasoconstrictor that induces acute insulin resistance by reducing capillary recruitment without altering total blood flow (Rattigan et al., 1999), blocked the increased myogenic activity in response to insulin along with decreased glucose uptake in rat muscle (Newman et al., 2009). Likewise, a recent report observed decreased myogenic activity, but preserved endothelial activity, during transdermal insulin administration in severely obese adolescents showing moderate hyperinsulinemia and insulin resistance (Montero et al., 2013a). Moreover, multiple regression analysis determined the myogenic activity as the primary predictor of insulin-mediated microvascular perfusion in the previous population (Montero et al., 2013c). Collectively, the study of vasomotion highlights the significance of the direct action of insulin in VSM, which if impaired, may lead to decreased insulin-mediated capillary recruitment and hence, less glucose and insulin availability for target tissues.

## Conclusions and perspectives

VSM emerges as a key player among the hemodynamic actions of insulin. A fine control of capillary recruitment may be accomplished by an insulin NO-dependent and/or –independent action in VSM, probably in terminal arterioles, which in turn regulates the interstitial delivery of nutrients and hormones. Therefore, a novel interpretation for vascular insulin resistance is suggested, indicating a central role for VSM dysfunction, which has indeed been recently established as a feature of the vascular pathology of insulin-resistant subjects (Montero et al., 2013b). Manipulation of insulin signaling pathways in VSM in pathophysiological states might thus improve vascular and, indirectly, metabolic functions of insulin. For that purpose, future research should be directed at elucidating mechanisms of VSM dysfunction impairing the hemodynamic actions of insulin, which at the moment are largely unexplored in humans.
